# Generalised linear regression GARMA model adopted in Denmark’s tourism industry

**DOI:** 10.1371/journal.pone.0329274

**Published:** 2025-08-22

**Authors:** Hongxuan Yan, Xingyu Yan, Luoyi Sun

**Affiliations:** 1 School of Mathematics and Physics, University of Science and Technology Beijing, Beijing, China; 2 School of Economics, Beijing Institute of Technology, Beijing, China; 3 North Automatic Control Technology Institute, Taiyuan, Shanxi, China; Cairo University, EGYPT

## Abstract

This paper investigates the characteristics of seasonality in the tourism industry. The Gegenbauer long memory and seasonal features are clearly clarified in Denmark’s tourism data. By plotting ACF and periodogram graphs, the pattern of long memory is investigated. A generalised linear regression GARMA (GLRGARMA) model and a generalised linear regression SARMA (GLRSARMA) model with an innovative function of explanatory variables is proposed to capture data features. Furthermore, the generalised Poisson (GP) distribution with over- equal- and under-dispersion is adopted to improve model flexibility. Eight sub-models are implemented with the number of rented hotel rooms data set to explore the best-performed model structure. The Bayesian approach is adopted to implement in-sample fitting and out-of-sample forecast studies. Several model selection criteria are adopted to evaluate model performances. Overall, GLRGARMA model is the best model to handle the time series with Gegenbauer long memory feature, especially in the tourism area. The explanatory variable with the periodic sponge effect will dramatically enhance model performances.

## Introduction

Tourism has become a vital economic pillar for nations globally. As consumers of goods and services, travelers drive demand that stimulates multiple industries, yielding employment opportunities, foreign currency inflows, and infrastructure development for host countries. Tourism stimulates the economic growth from different aspects, including directly boosting economic units from the tourism industry (housing, food, transportation, etc), creating new employment opportunities, bringing important revenues to the State budget in the form of taxes and fees and enhancing the developments of other sectors engaging in the accomplishment of the tourism product [1]. According to the statistical data from the WTO data for Tourism Sector including passenger Transportation Services (excluding freight), Travel Services, and the recreation portion of the Other Commercial Services sector [2], the proportion of the Tourism Sector is the sixth largest sector of the global economy and the largest Service Sector industry in the world [3]. Furthermore, the impacts of the development of tourism on industrial production have drawn great attention. [4] and [5] claimed that growing tourism will cause de-industralization, whereas [6] found that there is no evident negative impact of the development of tourism on the manufacturing industry in Thailand. Besides, tourism and hospitality increase the number of available jobs [7]. Consequently, tourism, a significant contributor to economic growth, needs to be investigated to reveal the dynamic mechanisms of tourism development through modeling and forecasting. The characteristics of development paths in tourism areas have also been underexplored.

Seasonality as a common characteristic has been widely found in the tourism industry [8]. Seasonality in tourism has traditionally been regarded as a major problem that needs to be overcome. [9] studied the characteristics of seasonality and developed a methodology to study this phenomenon.Recent evidence further highlights that seasonal variations in environmental conditions directly elevate pedestrian-accident risks in tourist areas by 18–24% [10]. [11] proposed quantitative solutions via financial portfolio theory to assist marketers in mitigating seasonal effects. [12] attempt to provide a rational framework for tourism seasonality by analysing the main characteristics of these challenges. Furthermore, in terms of the quantitative analysis method, [13] investigated the causalities of seasonality using a mixed effects panel data model for the main tourist destinations in the world. [14] proposes a new index to measure the seasonality in tourism by analysing the pattern of seasonal swings, including seasonal amplitude and similarity. This method focuses on the ordinal and cyclical structures of seasonal variations.The application of this methodology revealed a statistically significant association between seasonal variations and spatial distribution patterns across European countries. [15] found a seasonal phenomenon describing wane and wax shifting between the industry sector and the service sector. This phenomenon, named the periodic sponge effect, plays a vital role in economic development and addressing the unemployment issue. Moreover, they further propose a periodic sponge effect index that describes a reversed cyclical relationship between two time series. This index is a quantitative measure to define the strength of a periodic sponge effect accurately.

In terms of seasonal time series models in the tourism area, [16] studied ARIMA modeling seasonality in tourism forecasting with two settings, such as one for modeling stochastic nonstationary seasonality and another for a constant seasonality with three seasonal dummies. Moreover, the out-of-sample forecasting performances were evaluated. [17] argued that the simple deterministic model with seasonal dummy variables and AR(1) disturbances have better forecast performance. [18] compared the performance of various econometric time-series models in forecasting seasonal tourism demand and found that the methods of seasonality treatment, such as the pre-test for seasonal unit roots, affect the forecasting performance of the models. [19] forecasted tourism demand with ARMA-based methods. [20] adopted the seasonal ARIMA model to forecast the monthly outbound tourism departures. [21] investigated the performance of combination forecasts in international tourism demand. [22] proposed a SARIMA model with non-linear methods to forecast a seasonal tourist with a structural break in the data. [23] evaluated the performance of the Holt-Winters and Seasonal ARIMA models for forecasting foreign tourist arrivals in India. [24] proposed a multi-series structural time series method with one variable to predict seasonal tourism demand.

The long memory phenomenon has been widely studied in many areas, which motivates researchers to analyze this non-ignorable dependence between the present observation and all previous observations in a time series [25]. And the decay rate of this dependence can often be slower than exponential decay [26] provided a decent condition for a long memory stationary process using the autocorrelation function (ACF), denoted by ρ(j) for integers *j*, such that $\sum_{j=-\infty }^{\infty }\rho (j)=\infty$. Furthermore, [27] and [28] proposed the autoregressive fractionally integrated moving average (ARFIMA) model by incorporating a fractional differencing operator of certain order *d* (0<*d*<1/2) with the classical autoregressive integrated moving average (ARIMA) model. To capture the real-world cyclical phenomena with long-range dependence, [29] and [30] adopted the seasonal autoregressive fractionally integrated moving-average (SARFIMA) model. Moreover, to describe the long memory with an oscillatory pattern in many fields, [28] extended the ARFIMA model to Gegenbauer ARMA (GARMA) model by introducing Gegenbauer polynomial. To overcome the difficulties of applying the classical time series models to model, forecast and analyze time series in the form of counts, [31] constructed generalised linear ARMA (GLARMA) model by modifying the linear predictor into a ARMA time series structure in a generalised linear regression model. With the prevalence of seasonal discrete time series in many areas such as biology, finance and engineering, [32] extended GLARMA to Gegenbauer GLARMA (GLGARMA) model by replacing the ARMA structure in the linear predictor to Gegenbauer ARMA structure. Moreover, [33] further developed GLGARMA model by incorporating the period effect component and cohort effect component from the mortality model structure. Compared with classical Lee-Carter models, the model risk and associated forecast errors can be dramatically reduced.

## Contribution and structure

From a modeling perspective, our first contribution is to propose a generalised linear regression generalized autoregressive moving-average model (GLRGARMA) model and a generalised linear regression seasonal autoregressive integrated moving-average (GLRSARMA) model with an innovative function of explanatory variables in order to extend GLGARMA to incorporate relevant information for model fitting and forecast in the tourism area. Besides, the generalised Poisson (GP) distribution is adopted to accommodate over-, equal- and under-dispersion for certain tourism data. Model structures and properties of key components are precisely explained.

Our second contribution is to investigate the statistical features of the periodic sponge effect of tourism data. Especially, the pattern of long memory is examined. The analysis of the Hurst exponent, ACF plot and periodogram plot shows that Gegenbauer long memory features are presented in tourism data. Furthermore, the distinct characteristics between Gegenbauer long memory and seasonality are demonstrated to reveal that the GLRGARMA model is more suitable for modeling tourism data.

Our third contribution is the development of a Bayesian estimation framework for the proposed models, implemented via an efficient and user-friendly Rstan package. For the ML approach, the likelihood function is untractable because of involves very high dimensional integrals. Several monitors of convergence of posterior samples are discussed, such as the number of effective samples and $\hat{R}$ estimate. The criteria for modeling performance are also derived.

Our fourth contribution is to adopt both GLRGARMA and GLRSARMA to implement in-sample fitting and out-sample forecasts on Denmark’s economic data with an apparent periodic sponge effect. Overall, 8 nested sub-models are compared and evaluated using several well-known selection criteria. The models with Gegenbauer long memory components provide more accurate predictability than seasonal type models. Moreover, the explanatory variable with a stronger periodic sponge effect can dramatically enhance both in-sample fitting and out-sample forecast results.

The rest of the paper is structured as follows. The ’Periodic sponge effect’ section introduces and discusses this key economic phenomenon in Denmark’s tourism industry. Following this, the ’Generalised linear regression models with GARMA or SARMA latent processes’ section provides a foundational review of generalized linear models (GLMs) and long-memory time series frameworks for discrete data, followed by the formal introduction of the proposed GLRGARMA and GLRSARMA models, including their mean function specifications across eight distinct sub-models. The ’Bayesian inference’ section details the Bayesian estimation methodology for these models, alongside criteria for model selection and evaluation. In the ’Data analysis’ section, we demonstrate the practical utility of our framework through an empirical analysis of tourism data, reporting both in-sample fit metrics and out-of-sample forecasting performance. The ’Limitations and future work’ section discusses the constraints of the current study and potential directions for future research. Finally, the ’Conclusion’ section concludes with key insights and implications for future research.

## Periodic sponge effect

**Fig 1:**
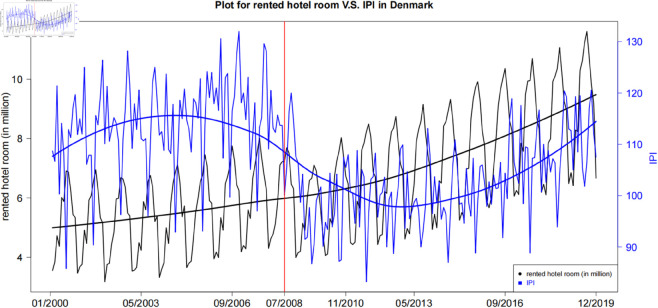
Time series plot for the number of rented hotel room (scaled in million) and IPI.

Denmark is a renowned and attractive Scandinavian country for visitors. Like other famous tourist attractions, there exists an obvious seasonal pattern in tourism. [15] proposed that there exists an apparent wane and wax shifting phenomenon between the developments of tourism and industry. Both tourism and industry follow totally opposite moving directions. This phenomenon is defined as the periodic sponge effect. Fig 1 shows the time series trend of the number of rented hotel rooms (in black) and IPI (in blue) from Jan 2000 to Dec 2019 in Denmark. Overall, both tourism and industry exhibit strong seasonality. The trend of IPI is counter-cyclical to the seasonal period of tourism. Details of data sets can be found in Section Data analysis.

There are two main drivers for the existence of the periodic sponge effect. The first one is the seasonal characteristic in tourism. The second one is the flexicurity labour market with flexible employment policies in Denmark, because there also exists a periodic labour mobility pattern between tourism and industry. Other causes of this effect, like tight immigration policy, higher level of unemployment assistance, and low barriers to entry tourism labour market, are also be discussed from various aspects. The existence of periodic sponge effect not only helps to understand the dynamic of the economic developments but also enhances the economy’s capacity to withstand risks. In many other places, the service sector has stirred concern about the possible drawbacks caused by the excessively strong tourism industry. However, [15] proved that the existence of the periodic sponge effect will avoid the Dutch Disease, which means that the increment of tourism does not harm economic development. Moverover, after experiencing a global disaster, such as great recession or global pandemic, the service sector with high return, low industry threshold and asset-light operation can recover rapidly to help other sectors, beacuse the existence of a periodic sponge effect means there exists a broad, smooth, and efficient transmission mechanism between the service industry and other industries, enabling it to cope with disaster shocks through flexible economic pattern. Furthermore, to address the unemployment issue in a global disaster, the periodic sponge effect provides enough buffer areas in the labour market. It allows a certain amount of labour flow and solves a sudden increase in the unemployment rate. Hence, to study the periodic sponge effect is vital to understand the dynamic of the economic developments. It provides a guideline for policymakers to predict labour flow and economic activities. In this paper, We will conduct a quantitative study on this phenomenon, deeply exploring its mathematical characteristics by constructing statistical models. Additionally, we will use time-series models to investigate its patterns and forecast the changes and trends.

## Generalised linear regression models with GARMA or SARMA latent processes

To model discrete time series with Gegenbauer long memory features, [32] extended the GLM to generalised linear generalized autoregressive moving-average model (GLGARMA) model, which combines the GLM and the GARMA time series model. The results from [32] show that generalised Poisson is consistently the best distribution choice compared with Poisson, NB and DP distributions. Furthermore, PD type models with higher flexibilities are often better than OD type models in both in-sample fitting and put-sample forecast. In terms of model structure, GLGARMA type model outperforms other types of models, including generalised autoregressive score (GAS) model [34], autoregressive conditional Poisson (ACP) model [35] and GLARFIMA model in both short and long memory data with or without obvious seasonal periodicity. In this study, since our models are extended from GLGARMA types model, based on the conclusions from [32], only PD type error term and GP distribution with Gegenbauer and seasonal periodic process are considered. Hence, generalised linear regression generalized autoregressive moving-average model (GLRGARMA) model and generalised linear regression seasonal autoregressive integrated moving-average (GLRSARMA) model are proposed to capture the periodic sponge effect in a time series.

**Definition 1** ( **GLRGARMA model**) *The GLGARMA model is a generalised state-space framework designed for discrete time series ${𝐘}_{1:T}\equiv (Y_{1},Y_{2},\ldots ,Y_{T})$, integrating an observation equation and latent state dynamics. To ensure strictly positive intensity parameters in count-valued processes, a canonical log-link function governs the conditional mean structure. For a stationary discrete time series with observed data filtration ${ℱ}_{1:t-1}=\sigma (Y_{1},Y_{2},\cdots ,Y_{t-1})$ with t∈[1,T], a GLGARMA model with order (p,d,q) is defined by*


$$Y_{t}|{ℱ}_{1:t-1},X_{1:t}\sim \text{GP}(\mu_{t},\nu ),$$

$$\Phi (B)\ln(\mu_{t})=\beta g(X_{t})+(1-2uB+B^{2}{)}^{-d}\Theta (B)\varepsilon_{t}$$

$$\varepsilon_{t}\overset{i.i.d}{\sim }N(0,\sigma^{2})$$

where $g(X_{t})\in {\mathbb{R}}^{W}$ are the function of explanatory variables representing the entire feature state at time *t* with regressors *X*_*t*,*w*_ for t∈[1,T] and w∈[1,W]. $\beta \in {\mathbb{R}}^{W}$ is a parameter vector that describes the relationship between **Y** and **X**. The dispersion parameter is defined as ν∈(−1,1) for GP distribution. The Gegenbauer parameter |u|<1 controls the pattern of oscillation and the long memory parameter 0<*d*<1/2 determines the strength of long memory. *B* is the backshift operator, such that $BY_{t}=Y_{t-1}$ and


$$\Phi (B)=1-\phi_{1}B-\cdots -\phi_{p}B^{p}\;\text{and}\;\Theta (B)=1+\theta_{1}B+\cdots +\theta_{q}B^{q},$$

are the autoregressive and moving-average characteristic polynomials, respectively, with no common roots.

**Definition 2** ( **GLRSARMA model**) *The GLSARIMA model with order (p,s,q) is given by*


$$Y_{t}|{ℱ}_{1:t-1},X_{1:t}\sim \text{GP}(\mu_{t},\nu ),$$

$$\Phi (B)(1-\alpha B^{s})\ln\mu_{t}=\beta g(X_{t})+\Theta (B)(1-\gamma B^{s})\varepsilon_{t}$$

$$\varepsilon_{t}\overset{i.i.d}{\sim }N(0,\sigma^{2})$$

where (1–${\text{B}}^{\text{s}}$) is the standard integer seasonal difference operator with the integer seasonal period *S* that defines the frequency of the seasonal pattern.

In both GLRGARMA and GLRSARMA frameworks, explanatory variables interact with the latent processes via additive separation: $\beta g(X_{t})$ modulates the mean trend directly. It represents explanatory information at time t, characterising the strength of association between the explanatory variables and the response variable *Y*_*t*_. While GARMA/SARMA components focus on investigating fundamental oscillation features of data. These two components capture the long memory/seasonal dependencies of historical observations. Considering some special cases of $\beta g(X_{t})$, the GLRGARMA model and GLRSARMA model can be further divided into 8 different sub-models with their mean functions defined in Table 1.

**Table 1:** 8 different sub-models where model 1,2,3,4 are GLRGARMA models, model 5,6,7,8 are GLRSARMA models.

Model	mean function
model 1	$\ln\mu_{t}=\beta_{0}+\beta_{1}X_{1}+{\left(1-2uB+B^{2}\right)}^{-d}\varepsilon_{t}$
model 2	$\ln\mu_{t}=\beta_{0}+\beta_{1}X_{1}+\beta_{2}X_{2}+{\left(1-2uB+B^{2}\right)}^{-d}\varepsilon_{t}$
model 3	$\ln\mu_{t}={\left(1-2uB+B^{2}\right)}^{-d}X+\varepsilon_{t}$
model 4	$\ln\mu_{t}={\left(1-2uB+B^{2}\right)}^{-d}X+{\left(1-2uB+B^{2}\right)}^{-d}\varepsilon_{t}$
model 5	$\ln\mu_{t}=\beta_{0}+\beta_{1}X_{1}+\alpha \ln\mu_{t-s}+(1-\gamma B^{s})\varepsilon_{t}$
model 6	$\ln\mu_{t}=\beta_{1}X_{1}+\beta_{2}X_{2}+\ln\mu_{t-s}+(1-B^{s})\varepsilon_{t}$
model 7	$\ln\mu_{t}=(1-\delta B^{s})X+\alpha \ln\mu_{t-s}+\varepsilon_{t}$
model 8	$\ln\mu_{t}=(1-\delta B^{s})X+\alpha \ln\mu_{t-s}+(1-\gamma B^{s})\varepsilon_{t}$

Model 1 and model 5 are simple generalised linear regression models with a constant $\beta_{0}$ and a single explanatory variable $\beta_{1}X_{1}$. These models can be regarded as baseline models for comparison. There are two explanatory components in model 2 and model 6 to evaluate the improvement of incorporating multi-source information. For model 3 and model 7, to test the cointegration between the periodic features (Gegenaber long memory and seasonal pattern) and explanatory variable, the periodic component is introduced to explanatory variable structure. Model 4 and model 8 are compositions of GLRGARMA/GLRSARMA model and explanatory variable with periodic component.

For the data distributions, GP distribution has the pmf, mean and variance given by


$$f(y_{t};\mu_{t},\nu )=\mu_{t}(1-\nu )[\mu_{t}(1-\nu )+\nu y_{t}{]}^{y_{t}-1}e^{-\mu_{t}(1-\nu )-\nu y_{t}}/y_{t}!,\;\mu_{t}>0,\;-1\leq \nu <1,$$

$$𝔼(Y_{t})=\mu_{t}\;\text{and}\;\text{Var}(Y_{t})=\mu_{t}(1-\nu {)}^{-2},$$

respectively. Furthermore, the GP distribution is over-, under- and equi-dispersed when ν is greater than, less than and equal to 0, respectively. On the other hand, the pmf for the DP distribution is given by


$$\tilde{f}(y_{t};\mu_{t},\nu )=c(\nu ,\mu_{t})f(y_{t};\mu_{t},\nu ),\;\mu_{t}>0,\;\nu >0,$$

where the normalizing term $c(\nu ,\mu_{t})$, given by [36], is


$$\frac{1}{c(\nu ,\mu_{t})}=\sum_{y=0}^{\infty }f(y_{t};\mu_{t},\nu )\approx 1+\frac{1-\nu }{12\mu_{t}\nu }\left(1+\frac{1}{\mu_{t}\nu }\right).$$

Because of the complicated structure of $c(\nu ,\mu_{t})$, some properties of the DP distribution are difficult to derive. The unnormalised pmf, mean and variance are given by


$$f(y_{t};\mu_{t},\nu )=(\nu^{1/2}e^{-\nu \mu_{t}})\left(\frac{e^{-y_{t}}y_{t}^{y_{t}}}{y_{t}!}\right){\left(\frac{e\mu_{t}}{y_{t}}\right)}^{\nu y_{t}},$$

$$𝔼(Y_{t})\approx \mu_{t}\;\text{and}\;\;\text{Var}(Y_{t})\approx \frac{\mu_{t}}{\nu },$$

respectively. The DP distribution is over-, under- and equi-dispersed when ν is less than, greater than and equal to 1 respectively.

### Mean functions

The key component of GLRGARMA model in the mean functions $\mu_{t}$ is based on a long memory process structure. There are two typical types of long memory model structure, the autoregressive fractionally integrated moving average (ARFIMA) model class and the generalised form of ARFIMA called Gegenbauer autoregressive integrated moving average (GARMA) model. [27] and [28] extended the classical ARIMA model to the ARFIMA model, which describe a long memory stationary process with integrated order d∈(0,1/2). The Gegenbauer ARMA (GARMA) framework [28] extends the generalized ARFIMA specification to accommodate time series with oscillatory-damped autocorrelation structures. This generalization leverages Gegenbauer polynomials to inherently capture long-memory patterns, providing a natural parametrization for cyclical persistence in autocorrelation functions (ACFs).

For d∈(0,1/2), the ARFIMA model exhibit long memory features. The short memory ARMA model is a special case of ARFIMA model with *d* = 0 where the long memory operator (1−*B*)^−2*d*^ = 1.

**Definition 3** ( **ARFIMA**) *Consider a stationary time series process with constant c∈ℝ, an ARFIMA model with order (p,d,q) is defined by*


$$\Phi (B)(\mu_{t}-c)=\Theta (B)(1-B{)}^{-2d}\varepsilon_{t},\;\varepsilon_{t}\overset{i.i.d}{\sim }N(0,\sigma_{\varepsilon }^{2}),$$

*where the long memory operator in ARFIMA model can be represented as*


$$(1-B{)}^{-d_{a}}=\sum_{j=0}^{\infty }\frac{\Gamma (j+d_{a})}{\Gamma (j+1)\Gamma (d_{a})}B^{j}=\sum_{j=0}^{\infty }\varphi_{j}B^{j}\;\text{with}\;d_{a}=2d.$$

For d∈(0,1/2) and u∈(−1,1), the GARMA model exhibits a long memory with an oscillatory pattern. The ARFIMA model is the special case of GARMA model with *u* = 1 (see [27]) such that the factor ${\left(1-2uB+B^{2}\right)}^{-d}$

**Definition 4** ( **GARMA**) *Consider a stationary time series process with constant c∈ℝ, a GARMA model with order (p,d,q) is defined by*


$$\Phi (B)(\mu_{t}-c)=\Theta (B)(1-2uB+B^{2}{)}^{-d}\varepsilon_{t}\equiv \Theta (B)\left(\sum_{j=0}^{\infty }\psi_{j}\varepsilon_{t-j}\right),\;\varepsilon_{t}\overset{i.i.d}{\sim }N(0,\sigma_{\varepsilon }^{2}),$$

*The Gegenbauer long memory operator in GARMA model can be represented as*


$$(1-2uB+B^{2}{)}^{-d}=\sum_{j=0}^{\infty }\psi_{j}\varepsilon_{t-j},$$

*and $\psi_{j}$ denote the coefficients of the generating function for the Gegenbauer polynomials ${\left(1-2uB+B^{2}\right)}^{-d}$* [37]. *These coefficients are formulated as*


$$\psi_{j}=\sum_{q=0}^{\left[j/2\right]}\frac{\left(-1{)}^{q}(2u{)}^{j-2q}\Gamma (d-q+j\right)}{q!(j-2q)!\Gamma (d)},$$

*where [*j*/2] represents the integral part of *j*/2.*

The coefficients $\psi_{j}$ in Eq (4) are functionally dependent on *d*, which controls the strength of long memory and the Gegenbauer parameter *u* that controls the oscillation of ACF [38]. The coefficients $\psi_{j}$ can be easily computed using the recursive formula:


$$\psi_{j}=2u\left(\frac{d-1}{j}+1\right)\psi_{j-1}-\left(2\frac{d-1}{j}+1\right)\psi_{j-2},$$

where the first three terms are $\psi_{0}=1$, $\psi_{1}=2du$ and $\psi_{2}=-d+2d(1+d)u^{2}$. Furthermore, [32] demonstrated that the bounds for the coefficients in the Gegenbauer polynomials $\psi_{j}$ are the coefficients of ARFIMA $\varphi_{j}$


$$\psi_{j}|\leq \frac{(2d{)}_{j}}{j!}=\varphi_{j}.$$

### Gagenbauer fractional differences versus seasonal difference in both time domain and frequency domain

In time series settings, the terms of long memory refers to the strength of statistical dependence, extended temporal dependence or persistence between lagged observations in a time series. And the rate that such lagged dependency decreases should be slower than exponential decay, which is the main feature in the long memory structure time series [25]. The Wold representation introduced by [39] states that

**Theorem 1.** Any zero-mean nondeterministic covariance-stationary process $Ys_{t\in \{1,2,3,\cdots ,T\}}$ can be expressed as

$$Y_{t}=c_{t}+\sum_{j=0}^{\infty }\psi_{j}\varepsilon_{t-j}=\Psi (B)\varepsilon_{t}+c_{t},\;\varepsilon_{t}\sim \text{WN}(0,\sigma^{2}),$$ (1)

where $\varepsilon_{t}$ and $\psi_{j}$ are uniquely defined and satisfy $\psi_{0}=1,\;\sum_{j=0}^{\infty }\psi_{j}^{2}<\infty$, $E(\varepsilon_{t})=0$, $E(\varepsilon_{t}^{2})=\sigma_{\varepsilon }^{2}$, $E(\varepsilon_{t}\varepsilon_{s})=0,\forall t,s$, the coefficients $\left\{c_{t};t\in \mathbb{Z}\right\}$ is a deterministic term with $E(c_{t},\varepsilon_{s})=0,\forall t,s$, WN stands for white noise.

Given a stationary time series process $Y_{1:T}\equiv (Y_{1},Y_{2},\ldots ,Y_{T})$ which admits Wold representation, with $Y_{1:T}\in {\left(\mathbb{N}\cup \{0\}\right)}^{T}$, [26] defined a condition for a long memory stationary process in terms of the divergence of the autocorrelation function (ACF) for *Y*_*t*_ and *Y*_*t* + *j*_ at lag *j*, such that


$$\underset{n\to \infty }{\text{lim}}\sum_{j=-n}^{n}|\rho (j)|\to \infty \;\text{where}\;\rho (j)=\frac{\text{Cov}(Y_{t},Y_{t+j})}{\sqrt{𝕍\text{ar}(Y_{t})𝕍\text{ar}(Y_{t+j})}}.$$

Parametric analysis of the autocorrelation function (ACF) in long-memory processes offers critical insights into their persistence characteristics, particularly through the interplay of fractional integration parameter *d* and Gegenbauer frequency parameter *u*. A notable case arises when *u* = −1 and 0<*d*<1/4 , where the ACF admits a closed-form representation:


$$\rho (j)=(-1{)}^{j}\frac{\Gamma (1-2d)\Gamma (j+2d)}{\Gamma (2d)\Gamma (j-2d+1)}\sim \text{constant}\cdot (-1{)}^{j}j^{4d-1}\text{, as}\;j\to \infty .$$

For ARFIMA(0,*d*,0) with *u* = 1 and 0<*d*<1/4, it is given asymptotically by,


$$\rho (j)=\frac{\Gamma (1-2d)\Gamma (j+2d)}{\Gamma (2d)\Gamma (j-2d+1)}\sim \text{constant}\cdot j^{4d-1}\text{, as}\;j\to \infty .$$

For the GARMA(0,*d*,0) process under the constraints |u|<1, 0<*d*<1/2, a closed-form expression for the autocorrelation function (ACF) remains analytically intractable [40]. However, its asymptotic behavior is characterized by:


$$\rho (j)\sim \text{constant}\cdot j^{2d-1}\sin(\pi d-j\lambda_{0})\text{, as}\;j\to \infty ,$$

where $\lambda_{0}=\cos^{-1}(u)$. The ACF plots of long-memory processes exhibit distinct patterns depending on parameters *d* and *u*.

According to the Szegö-Kolmogorov formula, the spectral density $f_{s}(\lambda )$ can be derived by taking the Fourier transform of autocovariance functions $\gamma_{\theta }(l-j)=(\Gamma_{\theta }{)}_{lj}$ and $I_{T}(\lambda_{k})$ can be derived by taking Discrete Fourier Transformation (𝔇(·)) of $Y_{t\in \{1,2,3,\cdots ,T\}}$ where $\lambda_{k}=\frac{2\pi k}{T}$ for $k=l-j=1,\cdots ,\left[\frac{T}{2}\right]$, [·] represents the integer part and only half of frequencies are enough to demonstrate the features because of symmetry. The spectral density is given by


$$f_{s}(\lambda )=\frac{1}{2\pi }\int_{-\infty }^{\infty }\gamma_{\theta }(k)\exp(-i\lambda k)dk,\;-\pi <\lambda <\pi .$$

In practice, the periodogram $I_{T}(\lambda )$ is usually employed as an estimator of the spectral density $f_{s}(\lambda )$. [?] stated that $I_{T}(\lambda )$ is unbiased but inconsistent estimator of $f_{s}(\lambda_{k})$ for a Gaussian white noise process $Y_{t\in \{1,2,3,\cdots ,T\}}$. The periodogram is given by


$$I_{T}(\lambda_{k})=\frac{1}{2\pi T}|𝔇(Y_{t\in \{1,2,...,T\}}){|}^{2}$$

$$=\frac{1}{2\pi T}{\left|\sum_{j=1}^{T}Y_{j}e^{-ij\lambda_{k}}\right|}^{2}$$

$$=Y_{r}^{2}(\lambda_{k})+Y_{i}^{2}(\lambda_{k}),$$

$$Y_{r}(\lambda_{k}):=\frac{1}{\sqrt{2\pi T}}\sum_{j=1}^{T}\cos(j\lambda_{k})Y_{j},$$

$$Y_{i}(\lambda_{k}):=\frac{1}{\sqrt{2\pi T}}\sum_{j=1}^{T}\sin(j\lambda_{k})Y_{j}.$$

For the ARFIMA(*p*,*d*,*q*) model

$$\Phi (B)(Y_{t}-c)=\Theta (B)(1-B{)}^{-d_{a}}\varepsilon_{t}\equiv \Theta (B)\left(\sum_{j=0}^{\infty }\varphi_{j}\varepsilon_{t-j}\right)\;\text{with}\;d_{a}=2d,$$ (2)

The spectral density function can be expressed as follow:


$$f_{s}(\lambda )=\frac{\sigma^{2}}{2\pi }{\left|1-e^{-i\lambda }\right|}^{-2d_{a}}\frac{{\left|\Theta (e^{-i\lambda })\right|}^{2}}{{\left|\Phi (e^{-i\lambda })\right|}^{2}}$$

$$=\frac{\sigma^{2}}{2\pi }{\left(2\sin\frac{\lambda }{2}\right)}^{-4d}\frac{{\left|\Theta (e^{-i\lambda })\right|}^{2}}{{\left|\Phi (e^{-i\lambda })\right|}^{2}}.$$

Furthermore, [28] showed that as λ→0,


$$f_{s}(\lambda )=\frac{\sigma^{2}}{2\pi }{\left(2\sin\frac{\lambda }{2}\right)}^{-4d}\frac{|\Theta (e^{-i\lambda }){|}^{2}}{|\Phi (e^{-i\lambda }){|}^{2}}\sim \frac{\sigma^{2}}{2\pi }\frac{|\Theta (1){|}^{2}}{|\Phi (1){|}^{2}}\lambda^{-4d}\sim \sigma^{2}\lambda^{-4d},$$

for 0<λ≤π. Consequently, $\lim_{\lambda \to 0}\lambda^{4d}f_{s}(\lambda )$ exists and is finite. For the special case of Φ(B)=Θ(B)=1, we have


$$f_{s}(\lambda )=\frac{\sigma^{2}}{2\pi }|1-e^{-i\lambda }{|}^{-4d}=\frac{\sigma^{2}}{2\pi }{\left(2\sin\frac{\lambda }{2}\right)}^{-4d}.$$

A useful generalised ARFIMA model called Gegenbauer ARMA (GARMA)(*p*,*d*,*q*) model proposed by [28] describes data showing slowly damping ACF with a cyclical pattern. The GARMA is given by

$$\Phi (B)(Y_{t}-c)=\Theta (B)(1-2uB+B^{2}{)}^{-d}\varepsilon_{t}\equiv \Theta (B)\left(\sum_{j=0}^{\infty }\Psi_{j}\varepsilon_{t-j}\right).$$ (3)

For the GARMA(*p*,*d*,*q*) process with long memory, the spectral density function was derived in [41] as follow:


$$f_{s}(\lambda )=\frac{\sigma^{2}}{2\pi }{\left|1-2u\exp(-i\lambda )+\exp(-2i\lambda )\right|}^{-2d}\frac{{\left|\Theta (e^{-i\lambda })\right|}^{2}}{{\left|\Phi (e^{-i\lambda })\right|}^{2}}$$

$$=\frac{\sigma^{2}}{2\pi }{\left(4\left|\sin\left(\frac{\lambda +\lambda_{0}}{2}\right)\sin\left(\frac{\lambda -\lambda_{0}}{2}\right)\right|\right)}^{-2d}\frac{|\Theta (e^{-i\lambda }){|}^{2}}{|\Phi (e^{-i\lambda }){|}^{2}},$$

where 0<*d*<1/2 and $u=\cos(\lambda_{0})$. Furthermore, it was demonstrated in [41] that the limiting behaviour of the spectral density function for the GARMA(*p*,*d*,*q*) model as $\lambda \to \lambda_{0}$ is


$$f_{s}(\lambda )=\frac{\sigma^{2}}{2\pi }\frac{|\Theta (e^{-i\lambda }){|}^{2}}{|\Phi (e^{-i\lambda }){|}^{2}}{\left(4\left|\sin\left(\frac{\lambda +\lambda_{0}}{2}\right)\sin\left(\frac{\lambda -\lambda_{0}}{2}\right)\right|\right)}^{-2d}$$

$$\sim \frac{\sigma^{2}}{2\pi }\frac{|\Theta (\lambda_{0}){|}^{2}}{|\Phi (\lambda_{0}){|}^{2}}{\left(4\left|\sin(\lambda_{0})\right|\right)}^{-2d}(\lambda -\lambda_{0}{)}^{-2d}$$

$$\sim \sigma^{2}(\lambda -\lambda_{0}{)}^{-2d}.$$ (4)

Hence, $\lim_{\lambda \to \lambda_{0}}{\left(\lambda -\lambda_{0}\right)}^{2d}f_{s}(\lambda )$ exists and is finite. For the special case Φ(B)=Θ(B)=1, we have

$$f_{s}(\lambda )=\frac{\sigma^{2}}{2\pi }[4(\cos(\lambda )-\cos(\lambda_{0}){)}^{2}{]}^{-d}=\frac{\sigma^{2}}{2\pi }[4(\cos(\lambda )-u{)}^{2}{]}^{-d}.$$ (5)

The differences between the SARIMA model and GARMA model are demonstrated by [32]. They further clarified that there exists a clear distinct feature between seasonal oscillation and the oscillation that comes from a Gegenbauer long memory process. Their results further showed that the deseasonalisation cannot remove the oscillating behavior in the Gegenbauer long memory time series. To reveal the fundamental difference between Gagenbauer fractional differences and seasonal difference in tourism area, this study simulate two sets of data with oscillation period of 12 (1 year period for monthly data). For SARMA model, the period is set to 12 which is agree with our real world data. For GARMA data set, the long memory parameter is *d* = 0.49 and Gegenbauer parameter is*u* = 0.7, which is similar to the period of SARMA.

**Fig 2:**
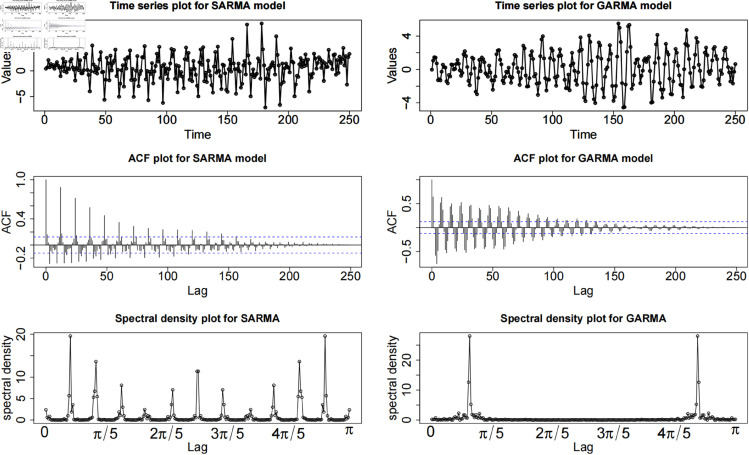
Simulation studies for SARMA and GARMA models.

The first row in Fig 2 shows the time series plot simulated by SARMA (left panel) and GARMA (right panel). According to the time series, the tendencies of SARMA and GARMA look very similar. It is hard to distinguish the nature between these two series. The ACF plots (second row) and periodogram (last row) plots are provided to analyze the fundamental characteristics between SARMA and GARMA. The ACF plot for SARMA shows damped periodic peaks with an overall short memory pattern. For GARMA, the ACF plot shows a typical Gegenbauer long memory ACF. There exists an oscillated long memory pattern. The periodogram plot as a representation of ACF in frequency domain can easily reveal the differences between SARMA and GARMA. The peaks for SARMA model in the periodogram plot are allocated in several places. These peaks must be located in 0, π/2 and *π* because the number of peaks represents the period, which can be regarded that these peaks chop the region [0,π] into 12 pieces. For GARMA model, the location of the peak represents the period of Gegenbauer long memory process, which can be interpreted as $\lambda =\cos^{-1}(u)$ where *λ* is the location of the peak.

## Bayesian inference

In this study, we employ Bayesian inference to perform in-sample fitting and out-of-sample forecasting, leveraging Bayes’ theorem to integrate prior structural knowledge into state-space formulations. This approach circumvents the computational challenges of evaluating marginal likelihood functions in partially observed models (e.g., high-dimensional integration over latent variables) [32]. Furthermore, our framework generates posterior predictive distributions for probabilistic forecasting, enabling nuanced interpretation of model characteristics. For instance, credible intervals for all parameters can be directly derived from their posterior distributions.

Let$y_{1:T}=\left(y_{1},y_{2},\ldots ,y_{T}\right)$denote a discrete-time series of non-negative integer observations($y_{t}\in {\mathbb{Z}}^{+}\cup \{0\}$)and $\vartheta^{*}$the parameter vector. Under the Bayesian paradigm, the posterior distribution of $\vartheta^{*}$ conditioned on $y_{1:T}$ is defined as:


$$\pi (\vartheta^{*}|y_{1:T})=\frac{f(y_{1:T}|\vartheta^{*})\;\pi (\vartheta^{*})}{\int f(y_{1:T}|\vartheta^{*})\;\pi (\vartheta^{*})\;d\vartheta^{*}}\propto f(y_{1:T}|\vartheta^{*})\pi (\vartheta^{*}),$$

which is proportional to the likelihood function $f(y_{1:T}|\vartheta^{*})$and the prior densities $\pi (\vartheta^{*})$, where priors are specified via empirical evidence or historical data. In the absence of prior knowledge, non-informative or reference priors can be adopted to preserve Bayesian objectivity.

### Bayesian model

Let $\vartheta^{*}=(\vartheta ,z)$ denote the vector of all model parameter $\vartheta =(\beta_{0},u,d,\theta_{j},\phi_{j},\sigma^{2},\nu )$ and state parameter $\varepsilon =\varepsilon_{1:T}=\left(\varepsilon_{1},\varepsilon_{2},\cdots ,\varepsilon_{T}\right)$, with each $\varepsilon_{t}\in \mathbb{R}$. For demonstrating purpose, both GLRGARMA model and GLRSARMA model with simple regression structure $\beta X_{t}$, *p* = 0 for Φ(B) and *q* = 0 for Θ(B) are proposed as examples. Hence, for GLRGARMA model, the latent process is $\ln(\mu_{t})=\beta_{0}\;+\;\beta (X_{t})\;+\;{\left(1\;-\;2uB\;+\;B^{2}\right)}^{-d}\varepsilon_{t}$ and the set of model parameter is $\vartheta =(\beta_{0},u,d,\beta ,\sigma^{2},\nu )$.

The priors π(ϑ) are defined as:


$$u\sim \text{U}(-1,1),\;d\sim \text{U}(0,1/2),\;\beta_{0}\sim \text{N}(0,\sigma_{c}^{2}),$$

$$\;\beta \sim \text{N}(0,\sigma_{\phi }^{2}),\;\sigma^{2}\sim \Gamma (a,b)\;\text{and}\;\nu \sim \text{U}(-1,1),$$

are adopted in which $\text{U}(a_{u},b_{u})$ denotes the uniform priors on the range $\left(a_{u},b_{u}\right)$ for the long memory parameters *u*, *d* and ν and Γ(a,b) denotes the gamma prior with shape and scale parameters *a* and *b* respectively for the scale parameter $\sigma^{2}$. The joint posterior distribution for the GLRGARMA(0,*d*,0) model with GP data distribution is


$$f(\vartheta^{*}|y_{1:T},x_{1:T})=f(y_{1:T}|\varepsilon_{1:T},x_{1:T},\vartheta )f(\varepsilon_{1:T}|\vartheta )\pi (\vartheta )\;\;\propto \;\prod_{t=1}^{T}\left[\frac{\exp(\beta_{0}+\beta (X_{t})+\sum_{j=0}^{\infty }\psi_{j}\varepsilon_{t-j})(1-\nu )[\exp(\beta_{0}+\beta (X_{t})+\sum_{j=0}^{\infty }\psi_{j}\varepsilon_{t-j})(1-\nu )+y_{t}\nu {]}^{y_{t}-1}}{\Gamma (y_{t}+1)\exp-y_{t}\nu -\exp(\beta_{0}+\beta (X_{t})+\sum_{j=0}^{\infty }\psi_{j}\varepsilon_{t-j})(1-\nu )}\;\right]\;\;\times \frac{1}{\sigma }\exp\;\left(\;-\frac{\varepsilon_{t}^{2}}{2\sigma^{2}}\right)\;\exp\;\left(-\frac{\beta_{0}^{2}}{2\sigma_{\beta_{0}}^{2}}\;\right)\;\cdot \;\left(-\frac{\beta^{2}}{2\sigma_{\beta }^{2}}\;\right)\;\cdot \;(\sigma^{2}{)}^{a-1}e^{-(\sigma^{2})b}I_{u}(-1,1)I_{d}(0,0.5)I_{\nu }(-1,1),\;$$

where the hyperparameters are set to be $\sigma_{c}^{2}=\sigma_{\beta }^{2}=10$, *a* = 3 and *b* = 1.

For GLRSARMA model, the latent process is $(1-B^{s})\ln\mu_{t}=\beta_{0}+\beta g(X_{t})+(1-B^{s})\varepsilon_{t}$ and the set of model parameter is $\vartheta =(\beta_{0},\beta ,\sigma^{2},\nu )$. The priors π(ϑ) are defined as:


$$\beta_{0}\sim \text{N}(0,\sigma_{\beta_{0}}^{2}),\;\beta \sim \text{N}(0,1),\;\gamma \sim \text{N}(0,1),\;\alpha \sim \text{N}(0,1),\;\sigma^{2}\sim \Gamma (a,b)\;\text{and}\;\nu \sim \text{U}(-1,1),\;$$

The joint posterior distribution for the GLRSARMA(0,*s*,0) model with GP data distribution is


$$f(\vartheta^{*}|y_{1:T},x_{1:T})=f(y_{1:T}|\varepsilon_{1:T},x_{1:T},\vartheta )f(\varepsilon_{1:T}|\vartheta )\pi (\vartheta )\;\;\propto \;\prod_{t=1}^{T}[\frac{\exp(\beta_{0}+\beta (X_{t})+\alpha \ln\mu_{t-s}+(1-\gamma B^{s})\varepsilon_{t-j})(1-\nu )}{\Gamma (y_{t}+1)\exp-y_{t}\nu -\exp(\beta_{0}+\beta (X_{t})+\alpha \ln\mu_{t-s}+(1-\gamma B^{s})\varepsilon_{t-j})(1-\nu )}\;.\times [\exp(\beta_{0}+\beta (X_{t})+\alpha \ln\mu_{t-s}+(1-\gamma B^{s})\varepsilon_{t-j})(1-\nu )+y_{t}\nu {]}^{y_{t}-1}]\;\times \frac{1}{\sigma }\exp\;\left(\;-\frac{\varepsilon_{t}^{2}}{2\sigma^{2}}\right)\;\exp\;\left(-\frac{\beta_{0}^{2}}{2\sigma_{\beta_{0}}^{2}}\;\right)\;\cdot \;\left(-\frac{\beta^{2}}{2\sigma_{\beta }^{2}}\;\right)\;\cdot \;(\sigma^{2}{)}^{a-1}e^{-(\sigma^{2})b}I_{\nu }(-1,1).\;$$

### Bayesian forecasting

A key strength of Bayesian forecasting lies in its capacity to generate full posterior predictive distributions for multi-step predictions. This framework implements *m*-step forecasts $y_{T+1:T+m}$, through iterative one-step-ahead forecasting, where each prediction $y_{T+s},\;s=1,\ldots ,m$ is conditioned on the expanding information set ${ℱ}_{s:T+s-1}$ and covariates $x_{1:T}$. The posterior predictive distribution for $y_{T+s},\;s=1,\ldots ,m$ is formally defined as:

$$\;f(y_{T+s}|{ℱ}_{s:T+s-1},x_{s:T+s})\;\;=\;\int \;\ldots \;\int \;f(y_{T+s}|\mu_{T+s},\vartheta ,{ℱ}_{s:T+s-1},x_{s:T+s})f(\mu_{T+s}|\mu_{s:T+s-1},\vartheta ,{ℱ}_{s:T+s-1},x_{s:T+s})\;f(\vartheta |{ℱ}_{s:T+s-1},x_{s:T+s})\;d\mu_{s:T+s}d\vartheta ,$$ (6)

And this integral is approximated by a Monte Carlo estimator using posterior samples:


$$\hat{f}(y_{T+s}|{ℱ}_{s:T+s-1},x_{s:T+s})=\frac{1}{L}\sum_{l=1}^{L}f(y_{T+s}|\mu_{s:T+s}^{\left(l\right)},\vartheta_{s}^{\left(l\right)},{ℱ}_{s:T+s-1},x_{s:T+s}).$$

In this analysis, we employ L=90,000 post-burn-in iterations per MCMC chain for each information window ${ℱ}_{s:T+s-1}$ and $x_{s:T+s}$. Here, $\mu_{s:T+s}^{\left(l\right)}$ and $\vartheta_{s}^{\left(l\right)}$represent the *l*-th posterior draws of the latent states $\mu_{s:T+s}$ and parameters ϑ, respectively. Beyond posterior predictive distributions, Bayesian inference further yields point estimators and predictive credible intervals for forecasts. Empirical Bayes forecasting, which conditions on in-sample posterior point estimates ($\tilde{\vartheta }$ and ${\tilde{\mu }}_{s:T+s}$) rather than marginalizing over parameter uncertainty, enhances computational efficiency [42]. Comparative analyses between frequentist and empirical Bayes forecasting methodologies are detailed in [43], with the latter omitting posterior integration steps in predictive distributions [32].


$${\hat{f}}^{\;\;EB}(y_{T+s}|{ℱ}_{s:T+s-1})=f(y_{T+s}|{\tilde{\mu }}_{s:T+s},\tilde{\vartheta_{s}},{ℱ}_{s:T+s-1}).$$

The point estimators $\tilde{\vartheta_{s}}$ and ${\tilde{\mu }}_{s:T+s}$ are typically derived from either the maximum a posteriori (MAP) estimate or the estimator minimizing the posterior expected loss. The MAP estimator generalizes the maximum likelihood (ML) principle under uninformative priors, as ${\tilde{\vartheta }}_{s}$ then coincides with the mode of the posterior distribution


$${\tilde{\vartheta }}_{s,MAP}=\underset{\vartheta_{s}}{\text{arg max}}f_{\vartheta_{s}}(\vartheta_{s}|{ℱ}_{s:T+s-1}).$$

Another Bayes estimator that minimises the posterior expected loss (PEL) is defined as


$${\tilde{\vartheta }}_{s,PEL}=\underset{\vartheta_{s}}{\text{argmin}}\;E(L(\vartheta_{s},{\tilde{\vartheta }}_{s}|{ℱ}_{s:T+s-1})),$$

where $L(\vartheta_{s},{\tilde{\vartheta }}_{s}|{ℱ}_{s:T+s-1})$ is the loss function. One example is the commonly used minimum mean square error (MSE) estimator defined as


$${\tilde{\vartheta }}_{s,MSE}=\underset{\vartheta_{s}}{\text{argmin}}\;E([\vartheta_{s}-{\tilde{\vartheta }}_{s}{]}^{2}|{ℱ}_{s:T+s-1}),$$

where ${\tilde{\vartheta }}_{s,MSE}$ corresponds to the posterior mean $E(\vartheta_{s}|{ℱ}_{s:T+s-1})=\bar{\vartheta_{s}}$. If the minimum absolute error (AE) estimator $L(\vartheta_{s},\tilde{\vartheta_{s}})=|\vartheta_{s}\;-\;{\tilde{\vartheta }}_{s}|$ is used, it gives ${\tilde{\vartheta }}_{s,AE}=\vartheta_{s,0.5}$ which is the posterior median.

### Bayesian tool: Implementation with Rstan

The proposed Bayesian models are implemented via the Rstan package, which interfaces with the Stan probabilistic programming language (C++ backend) for efficient inference. Rstan employs Hamiltonian Monte Carlo (HMC) sampling [44,45], a Markov chain Monte Carlo (MCMC) variant [46] that supersedes conventional methods like random-walk Metropolis [46] and Gibbs sampling [47] in high-dimensional parameter spaces. HMC accelerates convergence by reparameterizing the sampling problem through Hamiltonian dynamics [48], leveraging gradient information to generate distant proposals. This mechanism circumvents the inefficient state-space exploration inherent to random-walk methods, which exhibit diffusive behaviour in complex posterior landscapes.

For the HMC sampler, to assess the dependence, precision and convergence of the posterior sample, three measures are reported in Rstan. The first measure is the *number of effective samples* which indicates dependence within a Monte Carlo sample. The second measure is the *Monte Carlo standard error* (MCSE)


$$\text{MCSE}=\frac{\text{posterior standard deviation}}{\sqrt{\text{number of effective samples}}},$$

which reports the error of estimation for the posterior mean. To monitor the convergence for *k* > 2 chains of length 2*n* each, [49] proposed $\hat{R}$ which is defined as


$$\hat{R}=\frac{\hat{V}}{W}\cdot \frac{df}{df-2},$$

where

$$\hat{V}=\frac{n-1}{n}W+\frac{k+1}{kn}B,\;W=\sum_{i=1}^{k}\frac{s_{i}^{2}}{k},\;B=n\sum_{i=1}^{k}\frac{({\overset{―}{\vartheta }}_{i.}-{\overset{―}{\vartheta }}_{..}{)}^{2}}{k-1},\;df=\frac{2{\hat{V}}^{2}}{\hat{\text{V}}\text{ar}(\hat{V})},$$ (7)

$$\hat{\text{V}}\text{ar}(\hat{V})={\left(\frac{n-1}{n}\right)}^{2}\frac{1}{k}\hat{\text{V}}\text{ar}(s_{i}^{2})+{\left(\frac{k+1}{kn}\right)}^{2}\frac{2}{k-1}B^{2}+\;2\frac{\left(k+1)(n-1\right)}{kn^{2}}\cdot \frac{n}{k}[\hat{\text{C}}\text{ov}(s_{i}^{2},{\overset{―}{\vartheta }}_{i.}^{2})-2{\overset{―}{\vartheta }}_{..}\hat{\text{C}}\text{ov}(s_{i}^{2},{\overset{―}{\vartheta }}_{i.})],$$ (8)

*s*_*i*_ is the within-chain variance and $\vartheta_{ij}$ is the *j*-th parameter in chain *i*. If $\hat{R}$ is close to 1, the parameter ϑ has converged.

This analysis employs a single MCMC chain (*k* = 1), rendering between-chain variance *B* = 0 in Eqs (7) and (8). The chain executes 100,000 iterations, with the initial 10,000 iterations discarded as burn-in, yielding L=90,000 post-convergence draws (thinning interval = 1). Convergence diagnostics—including trace plots and $\hat{R}$ statistics—confirm all parameters satisfy $\hat{R}$ between 1.0000 and 1.0003, indicating stationarity and negligible between-chain variability. For both in-sample and out-of-sample analyses, effective sample sizes (ESS) range from 75,000 to 86,000 across parameters, reflecting moderate autocorrelation consistent with HMC’s sampling efficiency in high-dimensional spaces.

### Model selection and forecast performance

The performance of each model was evaluated using the deviance information criterion (DIC) [50], a widely adopted Bayesian model selection metric. DIC balances model fit and complexity, extending classical information criteria to hierarchical models and addressing limitations in traditional approaches. As a generalized version of Akaike’s Information Criterion (AIC), DIC extends its applicability to models incorporating informative prior distributions, particularly hierarchical Bayesian frameworks. This adaptation addresses a critical limitation of AIC in handling models with parameter constraints imposed through prior specification. Since informative priors inherently restrict parameter freedom, conventional parameter counting methods become inadequate for AIC calculations. DIC resolves this ambiguity by introducing a probabilistic estimate of the model’s effective dimensionality through the concept of "effective number of parameters."

The DIC can be calculated using the equation


$$\text{DIC}=\bar{D}+p_{D}=2\bar{D}-D({\bar{\vartheta }}_{x}),$$

where the deviance is defined as $D(\vartheta_{x})=-2\ln(f(y_{x}|\vartheta_{x}))$, $\bar{D}=E_{\vartheta_{x}|y_{x}}[-2\ln(f(y_{x}|\vartheta_{x}))]$ measures the model fit and the estimated number of parameters $p_{D}=\bar{D}-D({\bar{\vartheta }}_{x})$ measures model complexity [51]. To compare models, calculate the DIC for each model and choose the model with the lowest DIC. A lower DIC suggests a better fit given the model complexity.

The forecast performance of m-step-ahead predictions ${\hat{y}}_{x,t}$ (obtained via posterior mean or median estimators) relative to observed values *y*_*x*,*t*_ across *T* temporal intervals and gg demographic cohorts is quantitatively assessed through three fundamental error metrics: residuals $r_{x,t}=y_{x,t}-{\hat{y}}_{x,t}$ percentage errors $p_{x,t}=\frac{r_{x,t}}{y_{x,t}}\times 100$ and scaled errors $\epsilon_{x,t}$ as specified in *Eq* (11). Based on *r*_*x*,*t*_ and *p*_*x*,*t*_, three popular criteria, namely mean absolute error (MAE), root mean squared error (RMSE) and mean absolute percentage error (MAPE), are defined respectively below


$$\text{MAE}=\frac{1}{g}\sum_{x=1}^{g}\left[\frac{1}{m}\sum_{t=1}^{m}|r_{x,T+t}|\right],\;\text{RMSE}=\sqrt{\frac{1}{g}\sum_{x=1}^{g}\left[\frac{1}{m}\sum_{t=1}^{m}r_{x,T+t}^{2}\right]}$$

$$\text{and}\;\text{MAPE}=\frac{1}{g}\sum_{x=1}^{g}\left[\frac{1}{m}\sum_{t=1}^{m}|p_{x,T+t}|\right],$$ (9)

While relative errors *r*_*x*,*t*_ suffer from scale dependency, complicating cross-dataset comparisons, percentage errors *p*_*x*,*t*_—though scale-invariant—exhibit heightened sensitivity to near-zero observations. To address these limitations, we introduce the Mean Absolute Scaled Error (MASE) as a fourth evaluation criterion, defined by:

$$\text{MASE}=\frac{1}{g}\sum_{x=1}^{g}\left[\frac{1}{m}\sum_{t=1}^{m}|\epsilon_{x,T+t}|\right],$$ (10)

making use of the scaled errors

$$\epsilon_{x,T+t}=\frac{r_{x,T+t}}{\frac{1}{m-1}\sum_{t=2}^{m}|y_{x,T+t}-y_{x,T+t-1}|},$$ (11)

proposed by [52]. Furthermore, this analytical framework can be extended to evaluate posterior estimates ${\hat{\mu }}_{x,t}$ derived from mean or median estimators, where analogous error metrics—including residuals $r_{x,t}^{s}=\mu_{x,t}-{\hat{\mu }}_{x,t}$, percentage errors $p_{x,t}^{s}=\frac{r_{x,t}^{s}}{\mu_{x,t}}\times 100$ and scaled errors


$$\epsilon_{x,t}^{s}=\frac{r_{x,t}^{s}}{\frac{1}{m-1}\sum_{t=2}^{m}|\mu_{x,t}-\mu_{x,t-1}|},$$

can be systematically employed to establish parallel evaluation criteria for *μ* estimation, with MAE, RMSE, MAPE, and MASE directly computable using the unified computational formulations specified in Eqs (9) to (10).

## Data analysis

In this study, several economic data sets of Denmark are analysed because of the special political-economic features and the existence of periodic sponge effect. The Gini coefficient is the lowest (latest OECD figures from 2012) and there are numerous strong local companies with great competitiveness [53]. The data sets are obtained from [54] which is the central authority on Danish statistics.The complete dataset used in this analysis is provided in S1 File. This reliable data source is a state institution under the Ministry of Economic Affairs and the Interior. To ensure methodological consistency, the analysis utilizes 2000-2019 data exclusively, as Denmark implemented revised seasonal adjustment models in December 2020, rendering pre-February 2021 datasets incomparable with post-recalibration series [54]. From the macroeconomic scope point of view, Denmark has rescheduled their economic development from a predominantly agricultural country to a modern industrial country. Currently, the agriculture sector only contributes less than 2% of the overall GDP. The industrial base and services contribute around 18% and 76%, respectively. [54]. For the service sector, tourism is the most important component, which can be treated as a crucial representative indicator. Moreover, [15] shows the existence of the periodic sponge effect between tourism and manufacturing and their findings reveal the dynamic mechanism of the economic developments in Denmark. Furthermore, the flexicurity labour market with flexible employment policies is another special characteristic in Denmark. Employers maintain a very high level of flexibility, which means they can hire and fire whenever they want. On the other hand, the unemployment compensation is relatively high to guarantee a stable living standard for unemployed persons [55].

[15] also claimed that the flexicurity labour market with flexible employment policies can be regarded as another powerful driver to cause the periodic sponge effect. Consequently, on a micro-scale with monthly data, number of rented hotel rooms (scaled in a million), power production, industrial production index (IPI) and unemployed rate are adopted to investigate the dynamic mechanism of tourism sector statistics. In order to avoid the complex composition of IPI, Power production as a single statistical indicator of industrial production activities is also applied. The Power production is a physical quantity indicator that measures the total electricity consumption in the industrial sector, focusing on reflecting energy input in the production process. Sourced from power authorities, it is highly real-time and sensitive to energy-intensive industries, often used for monitoring short-term economic activity and analyzing high-energy-consuming sectors. The IPI is a relative index that comprehensively reflects changes in industrial output scale, covering all industries (including manufacturing, mining, etc.). Calculated from enterprise statistical data with a lag in publication, it focuses on output results and capacity utilization efficiency, suitable for medium-to-long-term trend research and macroeconomic cycle analysis. The two are closely related but may deviate due to factors such as industrial structure adjustment or changes in energy efficiency. For example, when the proportion of low-energy-consuming industries increases, the growth rate of the IPI may exceed that of power consumption, whereas the opposite may occur if energy-intensive industries dominate.

**Fig 3:**
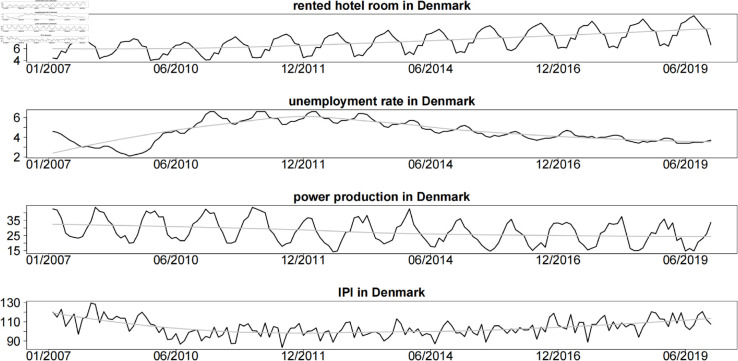
Time series plot of number of rented hotel rooms (scaled in a million), power production, industrial production index (IPI) and unemployed rate in a monthly scale.

Black curves in Fig 3 show a time series plot of rented hotel rooms (scaled in a million), power production, IPI and unemployed rate from January 2007 to June 2019. Grey lines are smoothed trends using a simple moving average approach. All of power production, IPI and unemployed rate show outstanding cyclical fluctuations with totally reversed period against number of rented hotel rooms. There exist strong periodic sponge effects in this group of data. The intensity of periodic sponge effect between number of rented hotel rooms and power production is strongest with an apparent reversed cyclical oscillation pattern. Moreover, the periodic sponge effect index between number of rented hotel rooms and power production is 0.89 which is the highest among these pairs [15].

### Empirical data analysis

The Hurst exponent *H*, also known as the index of long-range dependence, was proposed by [56]. It is a classical self-similarity parameter that measures the long memory feature in a time series [57]. Since it is robust with few assumptions about the underlying system, it has been widely applied to many fields [58]. A value of *H* in the range $\left(\frac{1}{2},1\right)$ indicates long memory in a time series, which means that a high value in the series will more likely be followed by another high value and such an effect is likely to maintain for a long period into the future. A value of $H=\frac{1}{2}$ can indicate a standard Brownian motion which is a short memory process. Furthermore, there exists a relationship between *d* and *H*, which is then given by *d* = *H*−0.5. Consequently, the estimator of the Hurst exponent *H* can approximate the long memory parameter *d* [32]. There exist various estimators of *H*, in this section, a well-known estimator called rescaled range analysis (R/S) is adopted to implement the following empirical studies.

[56] proposed the first Hurst exponent estimator using the rescaled range *R*/*S* analysis to measure the intensity of long-range dependence. Given a time series $Y_{t\in \{1,2,3,\cdots ,T\}}$, the sample mean and the standard deviation process are given by

$${\overset{―}{Y}}_{T}=\frac{1}{T}\sum_{j=1}^{T}Y_{j}\;\text{and}\;S_{t}=\sqrt{\frac{1}{t-1}\sum_{j=1}^{t}(X_{j}{)}^{2}},$$ (12)

where the mean adjusted series $X_{t}=Y_{t}-{\overset{―}{Y}}_{T}$. Then a cumulative sum series is given by $Z_{t}=\sum_{j=1}^{t}X_{j}$ and the cumulative range based on these sums is

$$R_{t}=\text{Max}\left(0,Z_{1},\cdots ,Z_{t}\right)-\text{Min}\left(0,Z_{1},\cdots ,Z_{t}\right).$$ (13)

An important proposition for the estimator of *H* was derived by [59].Consider a time series $Y_{t}\in R$ and define *S*_*t*_ and *R*_*t*_ in Eqs (12) and (13) respectively, then ∃  C∈R such that the following asymptotic property of the rescaled range *R*/*S* holds


$$[R/S](T)=\frac{1}{T}\sum_{t=1}^{T}R_{t}/S_{t}\sim CT^{H},\;\;\text{as}\;\;T\to \infty .$$

In addition, for small sample size *T*, the rescaled range *R*/*S* can also be approximated by following equation [60]


$$[R/S](T)=\left\{\begin{array}{c} \frac{T-1/2}{T}\frac{\Gamma ((T-1)/2)}{\sqrt{\pi }(T/2)}\sum_{j=1}^{T-1}\sqrt{\frac{T-j}{j}},\;\;\text{for}\;\;T\leq 340\\ \frac{T-1/2}{T}\frac{1}{\sqrt{T\pi /2}}\sum_{j=1}^{T-1}\sqrt{\frac{T-j}{j}},\;\;\text{for}\;\;T>340 \end{array}\right)$$

where the $\frac{T-1/2}{T}$ term was added by [61]. The *H* estimate can be obtained by a simple linear regression


logR/S(T)=logC+HlogT.

Hence, the definition for the estimator of *H* is given by the following equation

**Definition 5** ( **Estimator $\hat{H}$ by R/S**) *The estimator $\hat{H}$ based on the rescaled range *R*/*S* analysis is given by*

$${\hat{H}}_{R/S}=\frac{T(\sum_{t=1}^{T}\log R/S(t)\log t)-(\sum_{t=1}^{T}\log R/S(t))(\sum_{t=1}^{T}\log t)}{T(\sum_{t=1}^{T}(\log t{)}^{2})-(\sum_{t=1}^{T}\log t{)}^{2}}.$$ (14)

The empirical confidence interval of $\hat{H}$ given in Eq (14) with sample size *T* = 2^*N*^ [62] is


(0.5−exp(−7.33log(logN)+4.21),exp(−7.20log(logN)+4.04)+0.5).

**Fig 4:**
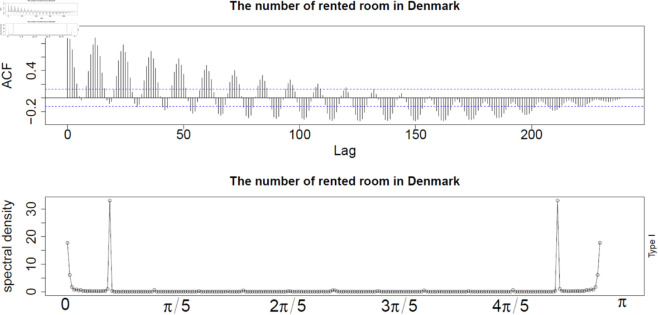
Plot of ACF and periodogram for rented hotel room numbers.

In this paper, the R package called pracma is adopted to estimate the value of *H* adopting the *R*/*S* analysis. For the data set of rented hotel room numbers in a month scale, the estimated Corrected R over S Hurst exponent is 0.908, which indicates that there exist a strong long memory in this data. Furthermore, according to the analytic plots for rented hotel room numbers in Fig 4, the class of long memory structure is a typical Gegenbauer long memory pattern with apparent oscillatory structure in ACF plot. For the periodogram plot, the peaks locate at non-zero position which aligns with the characteristics for Gegenbauer long memory type models.

### Model fitting

**Fig 5:**
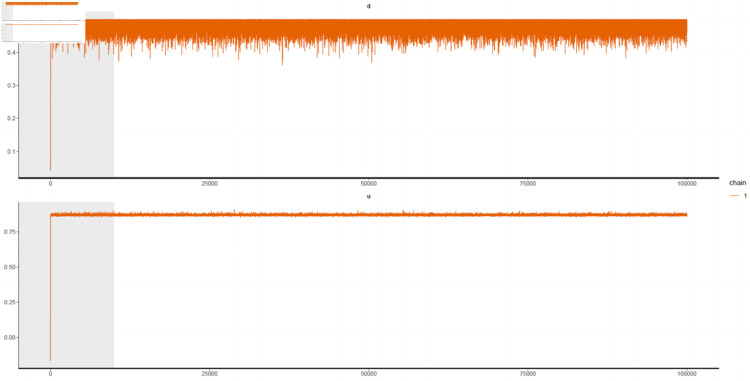
Trace plots for part of Model 1 parameters.

In this section, Model 1 to Model 8 are adopted to fit rented hotel room numbers in a month. Industrial production index (IPI), power production (PG) and unemployment rate (UR) are incorporated to improve model feasibility. The in-sample fitting performances of seasonal component and Gegenbauer long memory component are compared in this study. To monitor the convergence of Bayesian approach, the values of $\hat{R}$ for each estimator are between 1.0000 and 1.0003 and the number of effective sample is always more than 80,000. Fig 5 is an example of a convergence test, which reports the MCMC sample path for several key parameters for Model 1. According to these plots, the model parameters are properly estimated.

The goodness of fit for the eight models is evaluated by using DIC to select the best fitting model for each data set. Table 2 reported the DIC values of these models. The performance of the models incorporating Gegenbauer long memory or seasonal component on error terms are significantly better than the models with these components on the explanatory variables *X*. This indicates that the periodic historical information provided by the explanatory variables *X* will cause negative impacts on modeling performance. As contrast, the error terms only keep essential characteristics of previous knowledge for modeling. Furthermore, the performance of the model with seasonal component is similar to the long memory model since the differences in DIC values between both type models are very small. Consequently, for in-sample fitting study, Model 1, Model 2, Model 5 and Model 6 outperform other models.

**Table 2:** DIC results.

Model 1	X= IPI	X= UR	X= PG
DIC	1440.54	1285.48	825.4
Model 2	*X*_1_ = IPI, *X*_2_=PG	*X*_1_ = UR, *X*_2_=IPI	*X*_1_ = UR, *X*_2_=PG
DIC	863.48	1325.06	794.77
Model 3	*X* = IPI	*X* = UR	*X* = PG
DIC	2510.72	2367.663	2115.049
Model 4	*X* = IPI	*X* = UR	*X* = PG
DIC	2516.81	2416.19	2291.37
Model 5	*X* = IPI	*X* = UR	*X* = PG
DIC	1327.78	1287.67	825.87
Model 6	*X*_1_ = IPI, *X*_2_=PG	*X*_1_ = UR, *X*_2_=IPI	*X*_1_ = UR, *X*_2_=PG
DIC	847.93	1318.26	820.23
Model 7	*X* = IPI	*X* = UR	*X* = PG
DIC	2508.97	2397.63	2187.52
Model 8	*X* = IPI	*X* = UR	*X* = PG
DIC	2607.18	2417.82	2128.53

**Fig 6:**
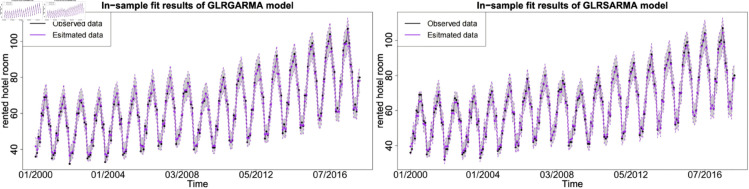
In-sample fitting plot for model 1 and model 5 with *X* = IPI.

**Fig 7:**
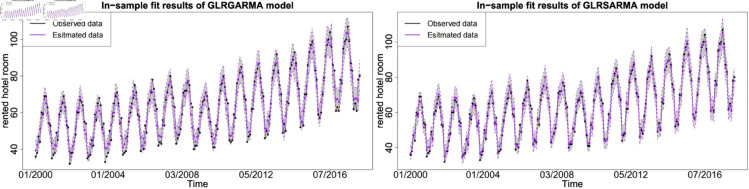
In-sample fitting plot for model 1 and model 5 with *X* = PG.

**Fig 8:**
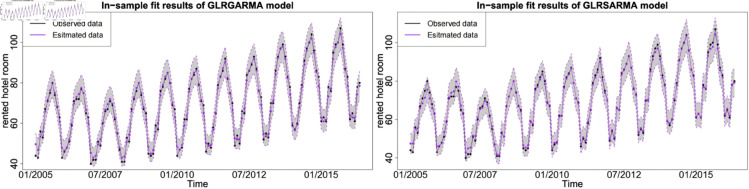
In-sample fitting plot for model 1 and model 5 with *X* = UR.

**Fig 9:**
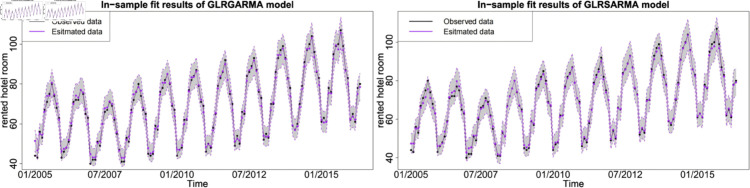
In-sample fitting plot for model 2 and model 6 with *X*_1_ = IPI and *X*_2_ = UR.

**Fig 10:**
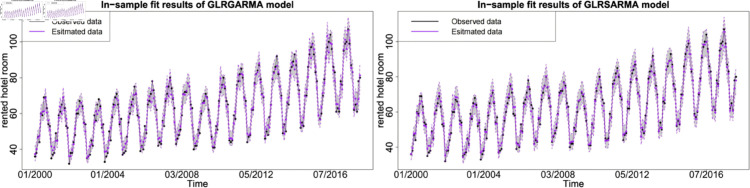
In-sample fitting plot for model 2 and model 6 with *X*_1_ = IPI and *X*_2_ = PG.

**Fig 11:**
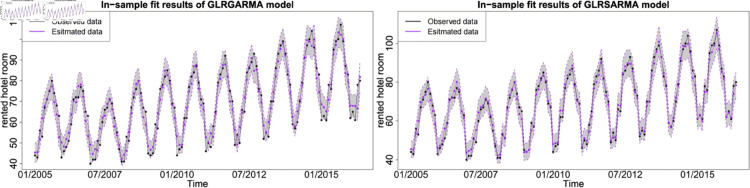
In-sample fitting plot for model 2 and model 6 with *X*_1_ = PG and *X*_2_ = UR.

The figures show the in-sample fit performance of selected best-performed models, which confirms that there is no significant evidence to claim long memory model superior than seasonal model in model fitting. Figs 6, 7 and 8 show the pairwise comparison of baseline model in in-sample fitting performance. The black dots with a dash line is the time series plot of observed data and the purple dots with dash line is the results generated from models. The grey area is the credible interval calculated from posterior samples. Both type models show reasonable and reliable fitting results. Furthermore, different information from explanatory variables will lead to distinct model performances. An explanatory variable with a significant periodic sponge effect, such as power production, will dramatically enhance model fitting results. Figs 9, 10 and 11 investigate the changes of model performances with an extra explanatory variable. The model fitting performance can be notably improved by introducing an explanatory variable with a stronger periodic sponge effect. Consequently, the periodic sponge effect plays a vital role in increasing model fitting accuracy.

### Model forecast

In this section, we calculate one-step ahead forecast for *m* = 20 time points based on the posterior predictive distributions and the posterior sample size of L=90,000. Only Model 1, Model 2, Model 5 and Model 6 are adopted in out-of-sample forecasting study because these models show reasonable in-sample fitting performance. The forecasts ${\hat{y}}_{t}$ are given by the posterior mean or median. To evaluate the forecast performance, three types of measures, namely residuals $r_{t}=y_{t}-{\hat{y}}_{t}$, percentage errors $p_{t}=\frac{r_{t}}{y_{t}}\times 100$ and scaled errors *q*_*t*_ are adopted. Based on *r*_*t*_ and *p*_*t*_, three popular criteria, namely the mean absolute error (MAE), root mean squared error (RMSE) and mean absolute percentage error (MAPE) are calculated in Table 3. Overall, Gegenbauer long memory models with smaller criteria values provide more accurate forecast results than seasonal models. Moreover, the forecast performance can be greatly improved by incorporating more explanatory variables.

**Table 3:** Comparison of models in forecasts with 95% Credible Intervals.

Model 1	X= IPI[CI]	X= UR[CI]	X= PG[CI]
MAE	15.43 [14.20, 16.80]	10.71 [9.80, 11.90]	8.08 [7.20, 9.10]
RMSE	16.75 [15.30, 18.40]	12.01 [10.90, 13.50]	9.59 [8.50, 10.80]
MAPE	0.16 [0.14, 0.18]	0.11 [0.10, 0.13]	0.09 [0.08, 0.10]
MASE	1.72 [1.58, 1.88]	1.34 [1.22, 1.48]	0.90 [0.82, 1.00]
Model 5	*X* = IPI[CI]	*X* = UR[CI]	*X* = PG[CI]
MAE	18.08 [16.50, 19.90]	12.01 [10.90, 13.40]	12.91 [11.70, 14.30]
RMSE	20.57 [18.70, 22.60]	13.16 [11.90, 14.70]	14.89 [13.50, 16.50]
MAPE	0.18 [0.16, 0.20]	0.12 [0.11, 0.14]	0.13 [0.11, 0.15]
MASE	2.02 [1.85, 2.20]	1.19 [1.08, 1.32]	1.44 [1.31, 1.59]
Model 2	*X*_1_ = IPI, *X*_2_=PG[CI]	*X*_1_ = IPI, *X*_2_=UR[CI]	*X*_1_ = UR, *X*_2_=PG[CI]
MAE	6.91 [6.20, 7.80]	9.91 [8.90, 11.10]	7.63 [6.80, 8.60]
RMSE	8.02 [7.10, 9.10]	11.27 [10.20, 12.60]	9.17 [8.20, 10.30]
MAPE	0.07 [0.06, 0.08]	0.10 [0.09, 0.11]	0.08 [0.07, 0.09]
MASE	0.77 [0.70, 0.85]	1.10 [1.00, 1.22]	0.85 [0.77, 0.94]
Model 6	*X*_1_ = IPI, *X*_2_=PG[CI]	*X*_1_ = IPI, *X*_2_=UR[CI]	*X*_1_ = UR, *X*_2_=PG
MAE	10.92 [9.80, 12.20]	9.92 [8.90, 11.10]	8.27 [7.40, 9.30]
RMSE	12.77 [11.50, 14.30]	11.54 [10.40, 12.90]	9.84 [8.80, 11.10]
MAPE	0.11 [0.10, 0.12]	0.10 [0.09, 0.11]	0.09 [0.08, 0.10]
MASE	1.22 [1.11, 1.35]	1.11 [1.01, 1.23]	0.92 [0.84, 1.02]

*Note: Values represent point estimates with 95% credible intervals in brackets, calculated from 90,000 MCMC posterior samples.*

**Fig 12:**
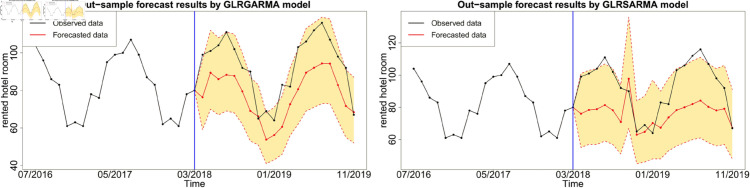
Out-of-sample forecasting plot for model 1 and model 5 with *X* = IPI.

**Fig 13:**
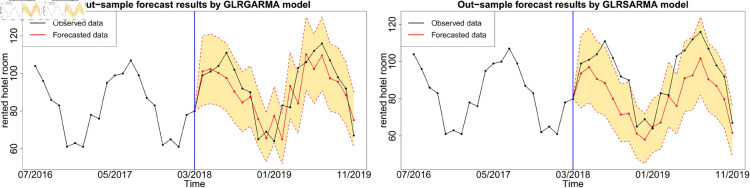
Out-of-sample forecasting plot for model 1 and model 5 with *X* = PG.

**Fig 14:**
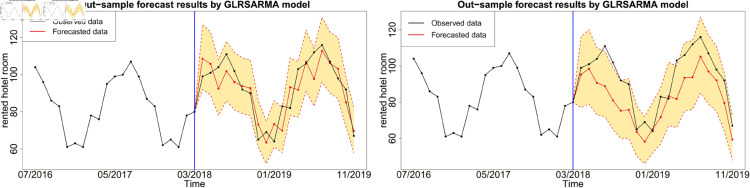
Out-of-sample forecasting plot for model 1 and model 5 with *X* = UR.

**Fig 15:**
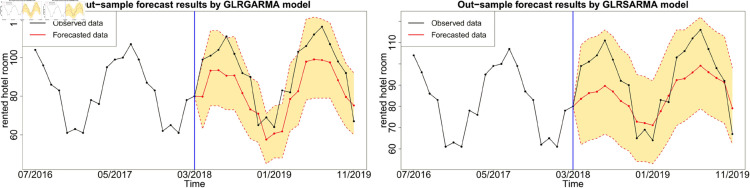
Out-of-sample forecasting plot for model 2 and model 6 with *X*_1_ = IPI and *X*_2_ = UR.

**Fig 16:**
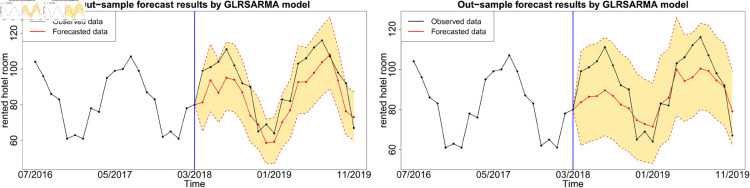
Out-of-sample forecasting plot for model 2 and model 6 with *X*_1_ = IPI and *X*_2_ = PG.

**Fig 17:**
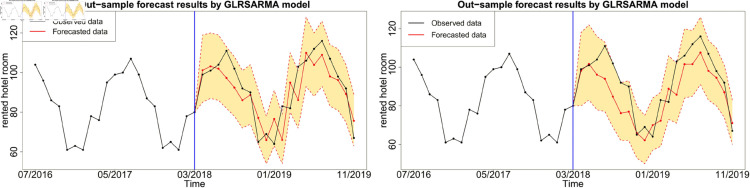
Out-of-sample forecasting plot for model 2 and model 6 with *X*_1_ = PG and *X*_2_ = UR.

Figures below show the forecast results, which agree with Table 3. Gegenbauer long memory type model should be the best choice in dealing with the number of rented hotel room data with Gegenbauer long memory features. Seasonal models cannot replace Gegenbauer long memory model since some fundamental features cannot be captured by a seasonal structure. Figs 12, 13 and 14 compare the predictability of the baseline model between Gegenbauer long memory type model and seasonal component type model. The black dots with dash line is the time series plot of observed data and the purple dots with dash line is the results forecasted from models. The grey area is the credible interval calculated from predictive posterior distribution. Similar to in-sample fitting study, the model incorporating the explanatory variable with a stronger periodic sponge effect produces more accurate forecasts. Figs 15, 16 and 17 describe the improvement of forecastability by adopting an extra explanatory variable with an intensive periodic sponge effect.

## Limitations and future work

There are some limitations to our current study. Modeling assumptions, such as linear relationships between variables, fixed distributional forms (e.g., Generalized Poisson), and strict separation of long memory from seasonality, may oversimplify real-world complexities like nonlinear interactions, time-varying heteroscedasticity, and dynamic feature couplings. Estimation procedures relying on Bayesian inference via Rstan face computational challenges in high-dimensional spaces, suffer from subjective prior specifications, and lack robustness due to single-model-selection criteria (e.g., DIC), while struggling to handle high-dimensional explanatory variables. To address these gaps, future work could incorporate flexible, data-driven methods like gradient-boosted machines (GBM), LSTM/Transformer networks, or Bayesian neural networks (BNNs) to capture nonlinear patterns and long-range dependencies. Additionally, integrating high-dimensional data through automated feature engineering, dimensionality reduction (e.g., autoencoders), and causal inference techniques (e.g., Double Machine Learning) would enhance predictive power, while model ensembles and distributed computing could improve computational efficiency and scalability for real-world tourism forecasting.

## Conclusion

This paper proposes a generalised linear regression structure with an innovative function of explanatory variables. Essential relevant information for modeling can be taken into consideration via explanatory variables. To capture the periodic oscillation features in a time series, both the seasonal component and Gegenbauer long memory component are incorporated to enhance model feasibility. Especially the fundamental differences between the seasonal component and Gegenabuer long memory component are distinguished. Moreover, the generalised Poisson (GP) distribution with over- equal- and under-dispersion is adopted to improve model flexibility. Furthermore, the existence of a periodic sponge effect among several key indices, including the number of rented hotel rooms, power production, IPI and unemployment rate, is discussed. The Gegenbauer long memory feature in the number of rented hotel rooms data is revealed. By plotting ACF and periodogram graphs, the long memory pattern is investigated. Overall, eight sub-models are implemented with the number of rented hotel rooms data to evaluate the model performance. The Bayesian approach is applied to implement in-sample fitting and out-of-sample forecast studies. Several model selection criteria are adopted to select the most feasible model. Overall, GLRGARMA model is evaluated to be the best model to handle the time series with Gegenbauer long memory feature, especially in the tourism area. The explanatory variable with a periodic sponge effect produces more accurate in-sample fitting results and out-sample forecasts.

## Supporting information

S1 File Denmark data.
